# Investigation of the removal kinetics, thermodynamics and adsorption mechanism of anionic textile dye, *Remazol Red RB*, with powder pumice, a sustainable adsorbent from waste water

**DOI:** 10.3389/fchem.2023.1156577

**Published:** 2023-06-02

**Authors:** Ahmet Gürses, Kübra Güneş, Elif Şahin, Metin Açıkyıldız

**Affiliations:** ^1^ Department of Chemistry, K.K. Education Faculty, Atatürk University, Erzurum, Türkiye; ^2^ Department of Science Education, K.M.R. Education Faculty, Kilis 7 Aralık University, Kilis, Türkiye

**Keywords:** adsorption, pumice, Remazol Red RB, pi-pi interactions, adsorption mechanism, dye removal

## Abstract

Excessive growth and abnormal use of dyes and water in the textile industry cause serious environmental problems, especially with excessive pollution of water bodies. Adsorption is an attractive, feasible, low-cost, highly efficient and sustainable technique in terms of green chemistry for the removal of pollutants from water. This study aims to investigate the removal kinetics, thermodynamics and adsorption mechanism of Remazol Red RB, which was chosen as a representative anionic reactive dye, from synthetic wastewater using powdered pumice, taking into account various experimental parameters such as initial dye concentration, adsorption time, temperature and pH. Moreover, to support the proposed adsorption mechanism, before and after adsorption of the samples, the Fourier transform infrared spectrophotometer (FTIR) spectra, X-ray powder diffraction (XRD) diffractograms and High resolution transmission electron microscopy (HRTEM) images were also taken and used. The results show that powder pumice can be an efficient adsorbent for anionic dye removal with a relatively high adsorption capacity of 38.90 mg/g, and it is very effective in 30–60 min in mild conditions. The experimental data showed a high agreement with the pseudo-second-order kinetic model and the Freundlich adsorption isotherm equation. In addition, thermodynamically, the process exhibited exothermic nature and standard isosteric enthalpy and entropy changes of −4.93 kJ/mol and 16.11 J/mol. K were calculated. It was determined that the adsorption mechanism was predominantly based on T-shaped pi-pi interactions and had physical characteristics.

## 1 Introduction

Industrial growth and rapid urbanization negatively affect people and the natural environment by causing serious environmental problems, especially with excessive pollution of water bodies. Particularly, there are intensive studies on effective methods and applications for the removal or disposal of toxic pollutants such as dyestuffs, heavy metal ions, plastics, medical wastes (Rajabi et al., 2022). Colorants are substances that are frequently used primarily for aesthetic and attractiveness purposes by using different dyeing techniques on various surfaces such as textiles, leather and paper products (Selvaraj et al., 2021). Dyes can be classified as cationic, anionic and non-ionic dyes besides various classifications (Anisuzzaman et al., 2022). Removal of anionic dyes is an urgent environmental problem that is difficult to solve as they are water soluble, have acidic properties and can give very bright colors in water (Dawood and Sen, 2014). Textile dyes reduce the light transmission of wastewater even at very low concentrations and increase the biological and chemical oxygen demands (BOD and COD), and moreover, they are toxic, carcinogenic or mutagenic for various organisms (Erdem and Ergun, 2020). Therefore, the treatment of industrial wastewater for decolorization has become an environmental obligation in terms of aesthetics and health (Hassan et al., 2020). In wastewater treatment, chemical, biological and physical methods are widely used and many techniques such as membrane filtration, advanced oxidation process, flocculation, coagulation, electrolysis, reverse osmosis, electrocoagulation, chemical precipitation and adsorption are applied at industrial scale (Gürses et al., 2003; Kim et al., 2004; Muntean et al., 2014; Donkadokula et al., 2020; Güneş et al., 2021; Gürses et al., 2021; Patel, 2022). Among these techniques, adsorption is very popular as a low-cost, easy-to-design and applicable technique in the treatment of industrial wastewater compared to other chemical, biological and physical methods (Muntean et al., 2017). Also, unlike other treatment methods such as coagulation, flocculation, oxidation, precipitation, electrolysis, reverse osmosis and membrane filtration, it does not produce harmful by-products that would pose environmental risks (Akdemir et al., 2022; Husien et al., 2022).

Pumice, which is a light, highly porous, low cost volcanic stone type with low apparent density in the range of 0.35–0.65 g/cm^3^, and high silica content, is one of the promising adsorbents that have been considered for use in various treatment technologies to remove pollutants in recent years (Veliev et al., 2006; Samarghandi et al., 2012; Alahyari Solokloei et al., 2022). It is also currently used in the construction industry, especially in the production of lightweight brick and concrete elements with heat and sound insulation properties (Ersoy et al., 2010). Due to its microporous structure and granularity, it is ideal for use in filled column filters for various contaminants (Quesada-Rodriguez et al., 2022). Due to the widespread use of reactive dyes in the textile industry, their annual growth rate is about four times that of other dyes (Zollinger, 2003). In particular, vinyl sulfone dyes, also known as remazol dyes, which are a class of reactive dyes, are used effectively in dyeing cotton, silk and wool (de Sousa et al., 2012; Ara et al., 2013). Azo dyes, such as Remazol Red RB, which are solid and mostly in salt form, usually have anionic colored components and their anionic character is due to the presence of a completely ionizable sulfonic group. Despite having a large molecular size and a high affinity for binding with cellulosic fibers, Remazol Red RB is less reactive than other dyes and therefore a relatively high amount of it remains in the dyeing bath and ends up in industrial wastewater (Ibrahim and Naghmash, 2023). Despite the widespread use and critical environmental effects in textile dyeing applications, not many studies were found on the removal and removal mechanisms of anionic dyes in the literature review. Therefore, this study focused on the investigation of the removal kinetics, thermodynamics and adsorption mechanism of Remazol Red RB, which was chosen as a representative anionic reactive dye, from synthetic wastewater using powder pumice, taking into account various experimental parameters such as initial dye concentration, adsorption time, temperature and pH of the suspension. In order to elucidate the adsorption mechanism of Remazol Red RB on the powder pumice surface, electro kinetic and static contact angle measurements of the samples obtained for various initial dye concentrations under certain conditions, as well as their before and after adsorption Fourier transform infrared spectrophotometer (FTIR) spectra, X-ray powder diffraction (XRD) diffractograms and High resolution transmission electron microscopy (HRTEM) images have been used.

## 2 Materials and methods

### 2.1 Materials

In this study, powder pumice with a specific surface area of 4.6 g/m^2^, which was supplied from Blokbims Co. in Turkey and considered as relatively waste due to its small particle size, was used as adsorbent. The mineralogical composition of the pumice used is shown in Table 1.

**TABLE 1:** The mineralogical composition of the pumice used.

Component	%	Component	%
SiO_2_	73.35	TiO_2_	0.08
Al_2_O_3_	12.88	MnO	0.05
CaO	0.77	Cr_2_O_3_	<0.01
MgO	0.08	SrO	0.01
Fe_2_O_3_	1.10	SO_3_	0.44
K_2_O	4.40	P_2_O_5_	0.01
Na_2_O	3.82	LOI	3.88

**FIGURE 1:**
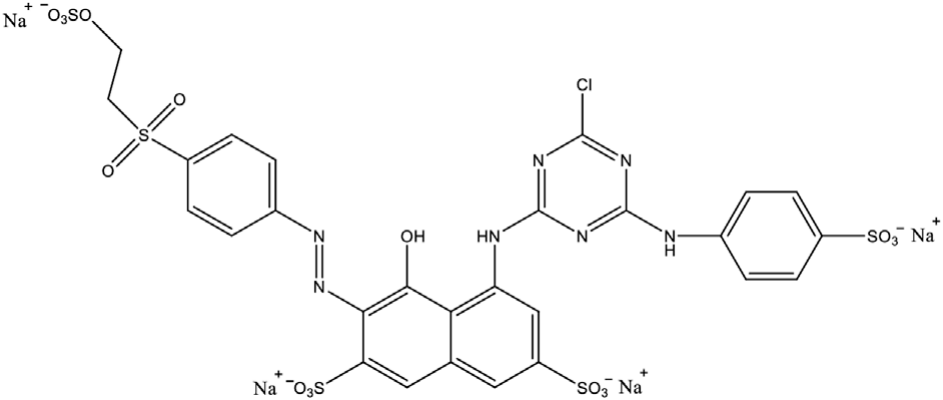
The chemical structure of Remazol Red RB.

Before use, raw pumice was dried in an oven at 110°C, ground and passed through the sieves in ASTM Standard, and then its fraction was taken in the range of 180–250 µm particle size, and stored in closed containers. Remazol Red RB (color index name: Reactive Red 198) is a commercially widely used reactive textile dye that is negatively charged in aqueous solution due to vinyl sulfone groups, and its chemical structure is given in Figure 1. In adsorption experiments, its aqueous solutions prepared at different concentrations were used as synthetic wastewater.

All chemicals used in this study were obtained from Merck Inc. and used without further purification.

### 2.2 Method

#### 2.2.1 Batch adsorption experiments

The adsorption of Remazol Red RB from aqueous solution on the powder pumice surface was carried out by batch adsorption experiments. For this, 0.2 g of powdered pumice was added to aqueous solutions of various initial dye concentrations (10, 20, 30, 40, 50, 60, 70 and 80 mg/L) in 100 mL flat bottom bottles. The balloons were shaken in a thermostatic shaker at 298 K, natural pH, and a stirring rate of 150 min^-1^ for various equilibrium adsorption times. At the end of the adsorption period, the mixture was centrifuged at 3,750 min^-1^ for 5 min and the equilibrium dye concentration in the supernatant was analyzed at 518 nm using UV-vis spectrophotometer (Shimadzu 1201 UV-Vis). The following equations were used to calculate the amount of dye adsorbed (q) and the removal ratio (R %) (Eqs 1, 2):

$$q\;\left(mg/g\right)=\frac{\left(C_{o}-C_{e}\right).V}{m}$$ (1)

$$R\left(\%\right)=\frac{\left(C_{o}-C_{e}\right)}{C_{0}}.100$$ (2)

where, C_o_ and C_e_ are initial and equilibrium dye concentrations (mg L^−1^), V total volume (L) and m, pumice mass (g), respectively.

In order to examine the change of adsorption efficiency and effectiveness with temperature, adsorption experiments were performed using 100 mg/L initial dye concentration and 0.2 g pumice for 60 min adsorption time at three different temperatures (25, 40 and 70°C). To investigate the effect of the initial pH, 0.2 g pumice was added to each 100 mL volume of the dye solution with an initial concentration of 100 mg/L adjusted to pHs of 2, 5, 8 and 11, and mixed at 25°C for 60 min. The pH of the solutions was adjusted with concentrated solutions of HCl and NaOH, and measurements were made using a pre-calibrated WTW inoLab pH meter (WTW Inc., Weilheim, Germany).

#### 2.2.2 Zeta potential measurements

Zeta potentials of solid particles in pumice/water suspensions taken from experiments carried out at 25°C for 60 min, as well as electrical conductivity values of suspensions were measured using Zeta Meter 3.0+.

#### 2.2.3 Static contact angle measurements

Static contact angle measurement was made using an optical goniometer (CAM-101, KSV Instruments, Finland). For this purpose, 0.4 g powder, raw and samples after adsorption, was pelleted under 1.06 tons/cm^2^ pressure, and the image of the drop formed on the solid with a 6 µL water droplet was taken, and the angle was calculated with the program taking into account the Young-Laplace equation.

#### 2.2.4 Spectroscopic and microscopic analyzes

Before and after adsorption, spectroscopic techniques such as XRD and FTIR, as well as microscopic techniques such as HRTEM were used to determine the possible distribution of the dye in the pumice matrix with the changes in structural crystallographic and functional groups of pumice, and thus to draw conclusions about the adsorption mechanism.

XRD diffractograms for raw pumice, dye and post-adsorption samples were taken using a PANalytical Empyrean X-ray Difractometer with Cu Kα1 (1.540 Å) radiation operating at 5 kV and 40 mA in the range of 2θ 9-90^◦^ and at the scanning speed of 4/min.

FTIR spectra the samples were also taken using Vertex 70 V FTIR spectrometer in the range of 4,000 to 400 cm^−1^ with an average of 100 scans and 1 cm^−1^ resolution.

HRTM images of the samples were taken using a HITACHI HT7700 high resolution transmission electron microscope (LaB6 filament) operating at 120.0 kV.

## 3 Results and discussion

### 3.1 Kinetic studies

**FIGURE 2:**
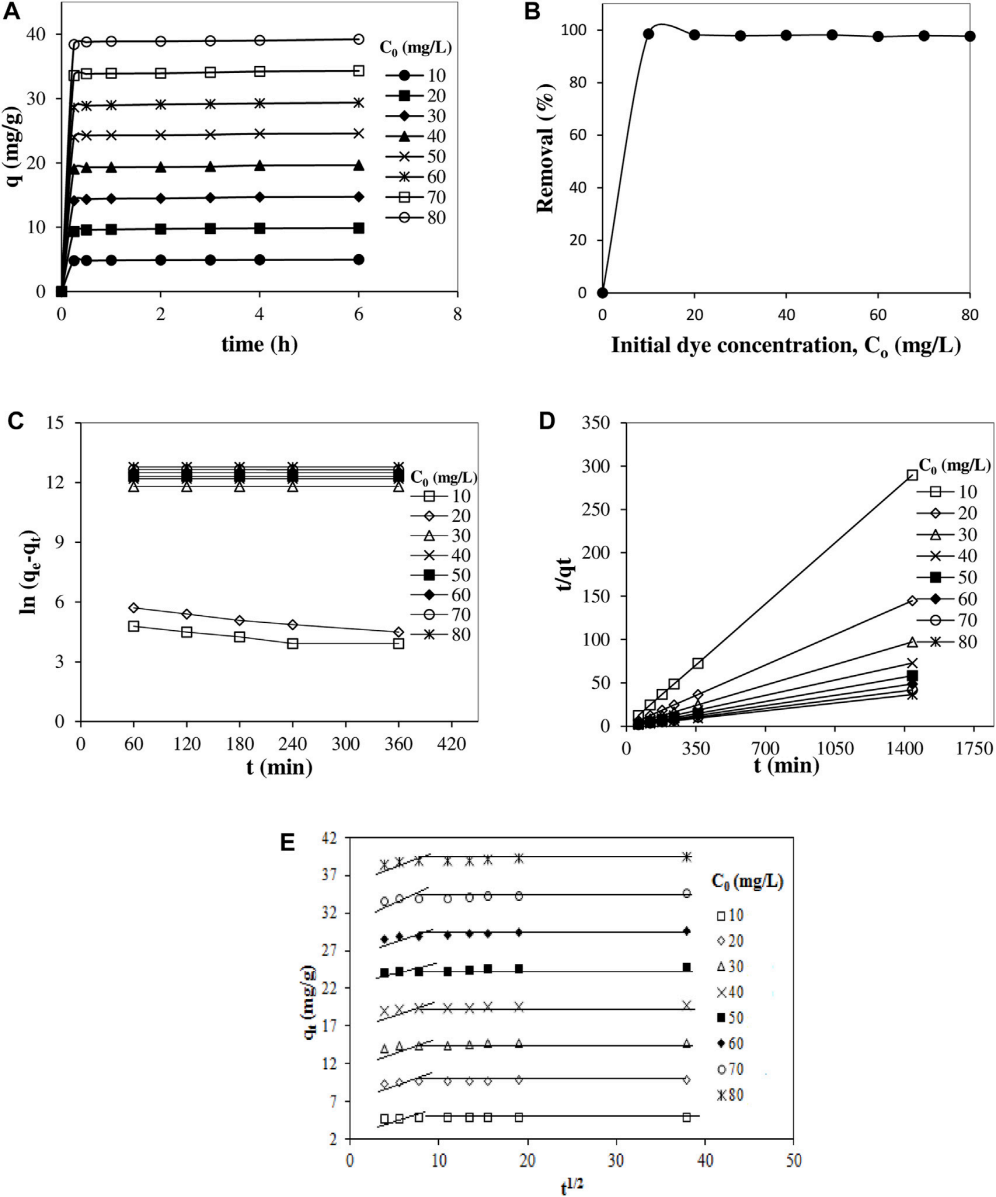
The variation of the amount adsorbed with adsorption time at various initial dye **(A)** and the variation of the removal ratios versus initial dye concentration for 60 min **(B)** and graphs of the pseudo-first order kinetic model **(C)**, the pseudo-second-order kinetic model **(D)** and the intra particle diffusion model kinetic model **(E)** for the adsorption of Remazol Red RB on powdered pumice (25°C, solid/liquid ratio: 0.2/100 g/mL, shaking speed: 150 min^−1^ and pH: natural pH:8).

The results obtained from the experiments examining the change of the amount of dye (Remazol Red RB) adsorbed by the powder pumice with the adsorption time are shown in Figure 2A, as a function of the initial dye concentration (10, 20, 30, 40, 50, 60, 70 and 80 mg/L). Also, the variation of % removal ratios versus initial dye concentration for 25°C is plotted in Figure 2B.

As can be seen from Figure 2A, the adsorbed dye amounts first increased rapidly with increasing adsorption time and remained almost constant after 30 min. On the other hand, it can be seen from the same figure that with the increasing initial dye concentration, the plateaus corresponding to adsorption saturations that occur in 30–60 min gradually increase. Accordingly, it can be said that the rather short duration of 30–60 min is sufficient for equilibrium adsorption, implying that the interactions that take place are physical in nature. Also, the rapid removal of the dye at the initial stage can be attributed to the presence of a large number of active sites on the pumice surface that are favourable for the interaction of dye molecules. These sites are probably hydrolysed Si-OH groups on the pumice surface, similar to the hydroxyl groups of cellulosic fibres with which the dye interacts very effectively. Since the change in the initial dye concentration did not appear to have any effect on the time required to reach saturation, to ensure that the adsorption equilibrium was reached, the equilibrium adsorption time was taken as 60 min in subsequent experiments.

Figure 2B shows that Remazol Red RB can be almost completely removed using powder pumice, with a very high removal rate in a fairly short adsorption time, and the removal ratios are not much affected by the initial dye concentration.

Studies on adsorption kinetics provide extremely important information about adsorption control mechanisms. Generally, adsorption can be controlled by external or film diffusion, pore diffusion, and adsorption on the pore surface, or their combination. For this reason, in order to predict the adsorption mechanism of the dye on the pumice surface, the compatibility of the experimental data with the pseudo-first-order, pseudo-second-order and intra particle diffusion models was investigated (Lagergren, 1898; Weber and Morris, 1963; Ho and McKay, 1999).

The linear forms of the equations of these models are given in Eqs 3, 4, 5, respectively.

$$\ln\;\left(q_{e}-q_{t}\right)=\ln\;q_{e}-k_{1}t$$ (3)

$$t/q_{t}=1/k_{2}{q_{e}}^{2}+t/q_{e}$$ (4)

$$q_{t}=k_{id}t^{1/2}+c$$ (5)

where q_e_ (mg/g) is the adsorbed amount at equilibrium, q_t_ (mg/g) is the adsorbed amount at time t (min), k_1_ (min^−1^) is the rate constant of the pseudo-first order model, k_2_ (g/mg min) is the rate constant of the pseudo-second-order model, k_id_ (mg/g min^1/2^) is the rate constant of the intra particle diffusion model, and c is a constant related to the thickness of the boundary layer. The graphs of pseudo-first-order, pseudo-second-order and intra particle diffusion models are given in Figures 2C, D, E, respectively, and the values of the calculated kinetic parameters and regression coefficients are shown in Tables 2, 3. From these figures and tables, it is seen that the kinetics of removal of dye from aqueous solution by adsorption on pumice can best be explained by the pseudo-second-order model. Therefore, it can be argued that there are very effective interactions between cationic dye ions and active sites on the pumice surface.

**TABLE 2:** Kinetic parameters of the pseudo-first order and the pseudo-second order kinetic models for the adsorption of Remazol Red RB on powdered pumice (25°C, solid/liquid ratio: 0.2/100 g/mL and pH: 8.0).

Initial concentration mg L^−1^	Temp. K	Pseudo-first-order		Pseudo-second-order			
		k_1_	*R* ^2^	q _e.exp_ (mg g ^−1^)	q _e.cal_ (mg g ^−1^)	k_2_ (g mg ^−1^ s ^−1^)	*R* ^2^
10	298	0.003	0.850	5.02	4.98	0.040	1.000
20	298	0.004	0.970	10.02	9.98	0.010	1.000
30	298	0.005	0.960	15.16	14.84	0.005	1.000
40	298	0.005	0.910	20.24	19.76	0.003	1.000
50	298	0.004	0.910	25.25	24.75	0.002	1.000
60	298	0.003	0.990	31.74	29.96	0.001	1.000
70	298	0.004	0.960	35.40	34.60	0.001	1.000
80	298	0.003	0.900	40.47	39.53	0.001	1.000

**TABLE 3:** Kinetic parameters of the intra particle diffusion kinetic model for the adsorption of Remazol Red RB on powdered pumice (25°C, solid/liquid ratio: 0.2/100 g/mL and pH: 8.0).

Initial concentration mg L^−1^	Temp. K	Intra particle diffusion (first part)			Intra particle diffusion (second part)		
		k_id,1_	C	*R* ^2^	k_id,2_	C	*R* ^2^
		mg s^−1/2^ g^−1^			mg s^−1/2^ g^−1^		
10	298	0.0112	4.75	0.983	0.0005	4.95	1.000
20	298	0.036	9.30	0.856	0.0064	9.7192	0.986
30	298	0.0415	14.03	0.888	0.0062	14.577	0.965
40	298	0.0338	19.02	0.888	0.0067	19.499	0.973
50	298	0.0326	23.97	0.843	0.0078	24.405	0.995
60	298	0.0442	28.55	0.929	0.0141	29.049	0.969
70	298	0.0426	33.531	0.9203	0.0145	34.024	0.987
80	298	0.042	38.434	0.81.11	0.0174	38.826	0.912

From Figure 2E, it is seen that the plot has two linear parts and does not pass through the origin. Accordingly, it can be claimed that intra particle diffusion is not the only step for rate control of the adsorption process and there may also be other control mechanisms (Gürses et al., 2006; Foo and Hameed, 2012). The first part of the plot can be related to the diffusion of dye through solution from the boundary layer to the outer surface of the powder pumice. The second part can be attributed to the intra particle diffusion (pore diffusion and adsorption on the pore surface) control. The slope of the linear portion indicates the rate of adsorption, and a lower slope corresponds to a slower adsorption (Oguntimein, 2015; El Maguana et al., 2020). Since the slope of the first part is higher than the slope of the second part, it can be argued that the dye molecules are rapidly adsorbed on the outer surface along the boundary layer instead of the pores of the pumice, and can only be adsorbed on the inner surface of the pumice pores when saturation is reached. The fact that the experimental data fit the pseudo-second-order kinetic model also supports these explanations, since pseudo-second-order kinetics indicate the existence of strong interactions or a high affinity between adsorbate and adsorbent.

### 3.2 Adsorption isotherms and the effect of temperature

Adsorption isotherm provides critical information about the nature of the interactions between the adsorbate molecules and the adsorbent surface at equilibrium, the adsorption capacity of the adsorbent, the adsorption efficiency and effectiveness, and the orientation of the adsorbate-molecules on the surface. These are also extremely important in terms of clarifying the adsorption mechanism.

**FIGURE 3:**
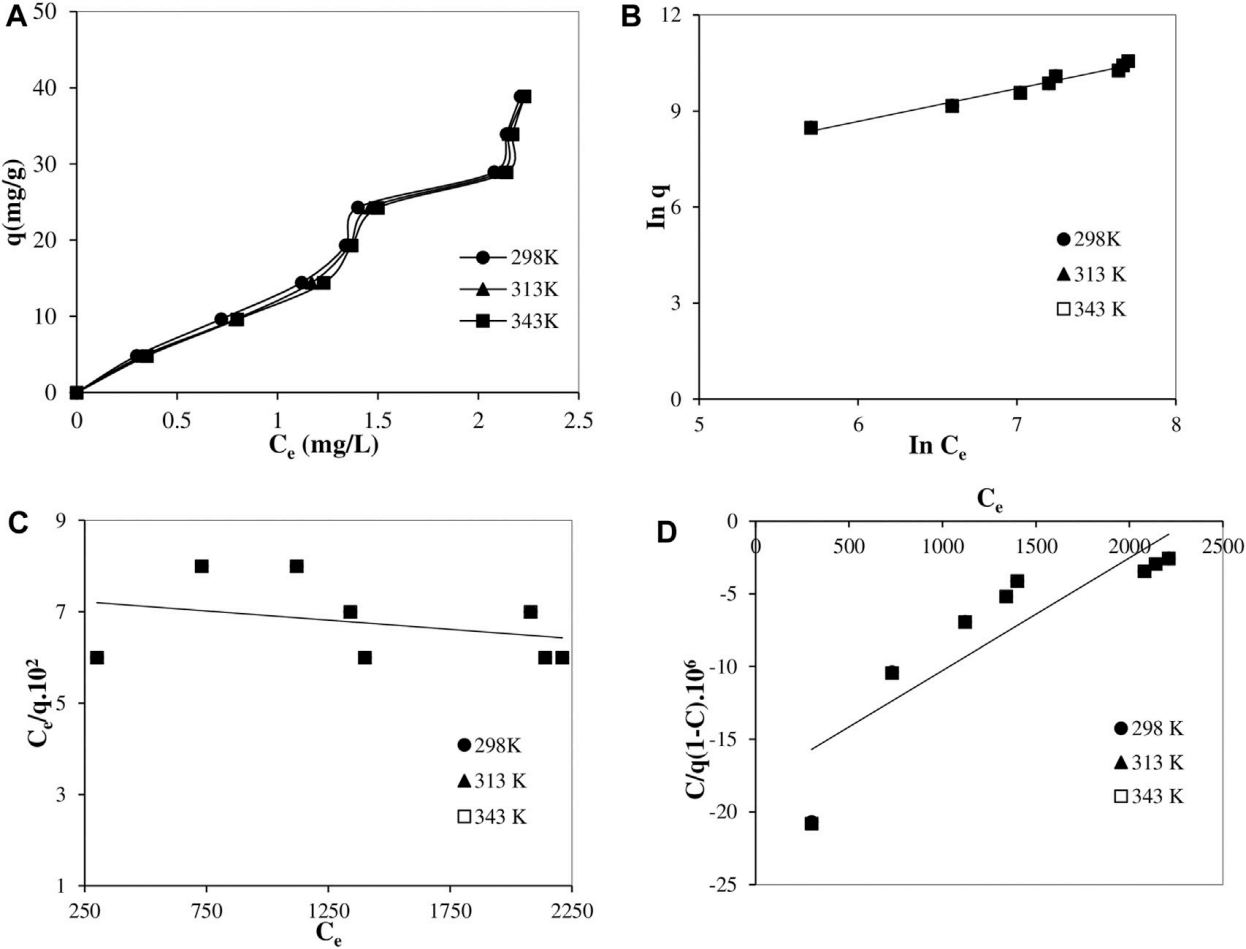
Experimental isotherm **(A)** and adsorption isotherms of Freundlich isotherm equation **(B)**, Langmuir isotherm model **(C)** and Brunauer–Emmett–Teller (BET) model **(D)** at 25, 40 and 70°C temperatures (Time:60 min; solid/liquid ratio: 0.2/100 g/mL, shaking speed: 150 min^−1^ and natural pH:8).

In this study, experimental adsorption isotherms for three different temperatures, 25, 40 and 70°C, were shown in Figure 3A. The compatibility of the experimental data with the Freundlich isotherm equation and Langmuir and Brunauer–Emmett–Teller (BET)isotherm models, which can be used to explain the adsorption of Remazol Red RB on the powder pumice surface in a different way, were also examined. Graphs drawn for the Langmuir, Brunauer–Emmett–Teller and (BET) isotherm models with the Freundlich isotherm equation were given in Figures 3B–D, respectively, and the isotherm parameters calculated for both them and Dubinin Radushkevich (D-R) isotherm model are shown in Table 4. The Freundlich isotherm equation is an empirical equation (El Maguana et al., 2019), and the BET adsorption isotherm model is based on multilayer adsorption assumptions. Whereas, Langmuir model assumes that there is no interaction between adsorbed ions or molecules, adsorption is localized and monolayer adsorption takes place on a homogeneous surface. The linear forms of this empirical equation and the two isotherm models are given in Eqs 6, 7, 8.

$$\ln q_{e}=\ln K_{F}+1/n\ln C_{e}$$ (6)

$$C_{e}/q_{e}=1/q_{m}K+C_{e}/q_{m}$$ (7)

$$C_{e}/q_{e}\left(1-C_{e}\right)=1/q_{m}k+\left(k-1\right)C_{e}/q_{m}k$$ (8)

where, C_e_ (mg/L) is equilibrium dye concentration, q_e_ (mg/g) is adsorbed amount in equilibrium, q_m_ (mg/g) is monolayer adsorption capacity, (1/n) and K_F_ are Freundlich constants (adsorption intensity or heterogeneity constant and capacity of adsorbent), K, Langmuir or adsorption equilibrium constant and k is BET parameter.

**TABLE 4:** Adsorption isotherm constants calculated by linear and nonlinear method at three temperatures for some adsorption models and equation.

	Linear method								
Temperature (K)		Langmuir				Freundlich			
		q_m_ (mg/g)	K (L/mg)	R^2^	Δq (%)	K_F_	n	R^2^	Δq (%)
298		−307.0	−0.046	0.080	63.1	12.75	0.977	0.971	11.98
313		−296.8	−0.047	0.085	62.3	12.61	0.976	0.971	12.00
343		−288.2	−0.048	0.089	62.5	12.43	0.974	0.971	11.94
BET						Dubinin-Radukevisch (D-R)			
		k	q_m_	R^2^	Δq (%)	q_m_ (mg/g)	β (10^−7^)	R^2^	Δq (%)
298		0.999	−5582	0.778	87.9	30.36	1.53	0.825	89.0
313		0.999	−5567	0.779	88.0	30.36	1.39	0.824	89.0
343		0.999	−5549	0.778	89.0	30.33	1.16	0.825	89.0
Non-Linear Method									
Temperature (K)		Langmuir				Freundlich			
		q_m_ (mg/g)	K (L/mg)	R^2^	Δq (%)	K_F_	n	R^2^	Δq (%)
298		4.482	−1.052	—	128.4	5.32	0.875	0.954	13.64
313		4.481	−1.052	—	128.6	5.22	0.873	0.955	13.71
343		4.478	−1.052	—	128.7	5.16	0.872	0.954	13.75
BET						Dubinin-Radukevisch (D-R)			
		k	q_m_	R^2^	Δq (%)	q_m_ (mg/g)	β (10^–7^)	R^2^	Δq (%)
298		0.999	−9236	0.903	45.5	50.52	4.48	0.919	39.9
313		0.999	−9.218	0.903	45.6	50.58	4.07	0.920	40.0
343		0.999	−9.204	0.903	45.8	50.60	4.50	0.920	40.1

The linear form of the Dubinin-Radushkevich isotherm model (D-R) (Adusei et al., 2022), which is generally used to determine the porosity of the adsorbent and the free energy of adsorption, is given in Eq. 9.

$$\ln q_{e}=\ln q_{m}-K\varepsilon^{2}$$ (9)

where q_e_ is the equilibrium adsorbed amount of the adsorbed substance (mg g^−1^), q_m_ is the theoretical saturation capacity (mg g ^−1^), K is the activity coefficient related to the free energy of adsorption (mol^2^ kJ^−2^), ɛ is the Polanyi potential (kJ mol^−1^).

ɛ=RT⁡ln(⁡1+1Ce) (10)

where, R is universal gas constant (8.314 J mol^−1^ K^−1^), T is absolute temperature (K), C_e_ is equilibrium concentration of adsorbate (mg L^−1^).

In order to quantitatively compare the applicability of the investigated both isotherm equation and models, the normalized standard deviation Δq values were also calculated (Eq. 11) and the obtained values are given in Table 4 together with the regression coefficients (*R* ^2^) (Açışlı et al., 2022).

$$∆q\;\left(\%\right)=\sqrt{\sum \left[q_{t,\exp}-q_{t,cal}\right]2/q_{t,\exp}/\left(n-1\right)}\;.100$$ (11)

where n is the number of experimental data, q_t,exp_ is the experimental adsorption capacity and q_t,cal_ is the calculated adsorption capacity.

From Figure 3A it can be seen that with a similar trend for all three temperatures, with increasing equilibrium dye concentration, the adsorbed amounts increase with a relatively low yield up to medium equilibrium dye concentrations and reach a plateau. A higher adsorption efficiency is then observed with further increase in equilibrium dye concentrations. Again, this graph shows that although there is a partial slight decrease in the adsorbed amounts with increasing temperature, the adsorption efficiency and effectiveness were generally not affected much by the temperature.

It can be seen from Table 4 that the adsorption of Remazol Red RB on powder pumice conforms to the Freundlich isotherm equation with a high correlation coefficient (*R* ^2^). This indicates the existence of a heterogeneous surface in terms of adsorption, which is consistent with the results from the kinetic analysis, and the effectiveness of dye-surface interaction with partially low efficiency and multi-layer interactions with a higher efficiency, in parallel with the proposed adsorption mechanisms.

**FIGURE 4:**
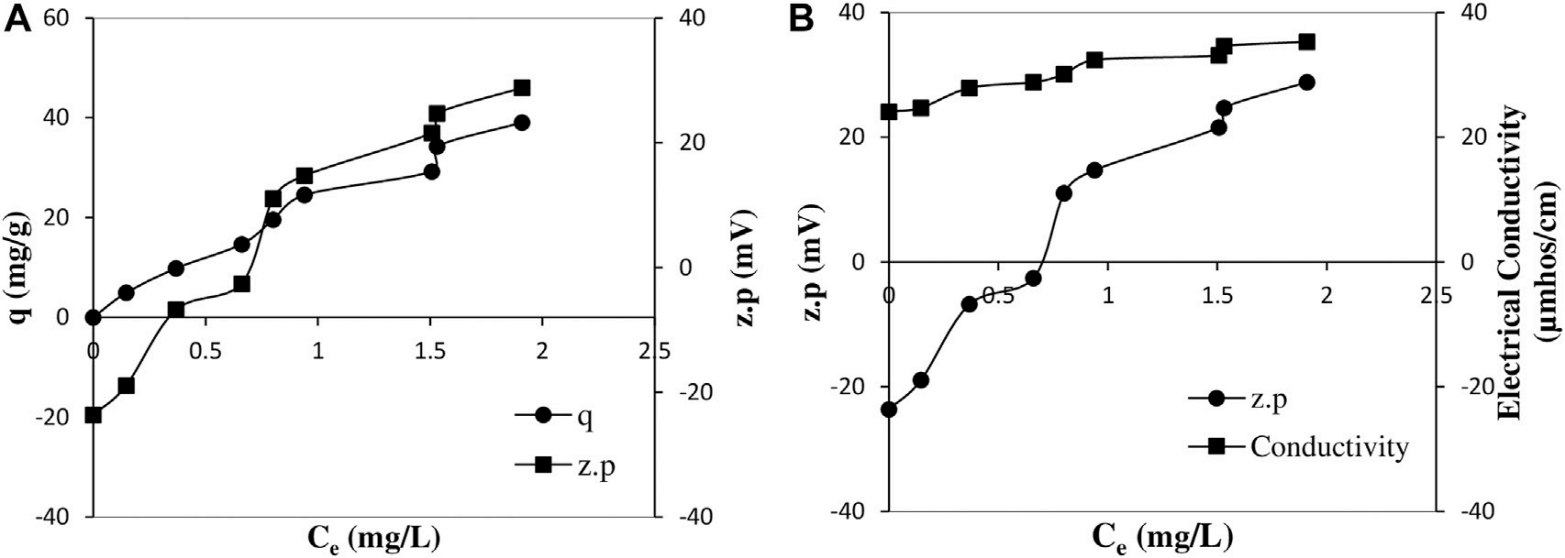
Variation of adsorbed dye amounts and zeta potential values of pumice particles with equilibrium dye concentrations **(A)** and variation of zeta potential values of pumice particles and electrical conductivity values of suspensions with equilibrium dye concentrations **(B)** at 25°C (Time:60 min; solid/liquid ratio: 0.2/100 g/mL, shaking speed: 150 min^−1^ and natural pH:8).

In addition, in order to evaluate the nature of the interactions between adsorbate and adsorbent and the orientation of dye ions on the surface, graphs showing the changes in both the zeta potential values of the pumice particles and their adsorbed dye amounts and the zeta potentials and the electrical conductivity values of their suspensions with the equilibrium dye concentration at 25°C were also drawn, and are given in Figures 4A, B, respectively.

Interestingly, it can be seen from Figures 4A, B that the variation of the zeta potential and electrical conductivities with the equilibrium dye concentration is extremely similar to the trend of change in the curve of the equilibrium dye concentration and variation of the adsorbed amounts. The zeta potential values, which were negative at low equilibrium dye concentrations, became positive, although not very high, with increasing equilibrium dye concentration. The similarity of the trend of change of the curve with that of the adsorbed amounts precisely implies the relationship with the adsorption mechanism. It can be said that hydrogen bonding interactions between the hydroxyl groups of the dye and the hydrolyzed Si-OH groups of pumice are effective in adsorption, predominantly at low equilibrium dye concentrations. The source of the partial positive zeta potential values observed at higher equilibrium dye concentrations may be due to the mineralogical composition of the pumice and thus possible hydrolysis or unequal dissolution of some oxides. For this reason, it can be claimed that in addition to the adsorption of dye ions through hydrogen bonding, additional adsorption takes place by the mechanism of ion pairing through the positively charged sites formed on the pumice surface due to the unequal adsorption of the divalent or trivalent ions originating from the pumice. The tendency of electrical conductivity values to increase first and then to remain relatively constant at higher concentrations with increasing equilibrium dye concentration also supports this claim. The sharp increase in adsorbed amounts observed with further increase in equilibrium dye concentration can be attributed to be predominant of non-covalent π–π interactions called π–π stacking as T-shaped or parallel-displaced, both of which are based on electrostatic attraction.

### 3.3 Adsorption thermodynamics

The nature and thermodynamics of dye adsorption on pumice powder were also evaluated by calculating the isosteric standard adsorption enthalpy (ΔH°) and entropy (ΔS°) values for a constant adsorbed amount of 4.85 mg/g using the isotherm data obtained at 25, 40 and 70°C. The calculations were made using Eqs 12, 13 with equilibrium dye concentrations at different temperatures corresponding to the same adsorbed amount.

$$d\;\left(\ln C_{e}\right)/d\;\left(1/T\right)={-\Delta H{}^{\circ}}_{ads.}/R$$ (12)

$$d\;\left(\ln C_{e}\right)/d\;\left(\ln T\right)={\Delta S{}^{\circ}}_{ads.}/R$$ (13)

Isosteric adsorption enthalpy, ΔH° _ads_ and isosteric adsorption entropy ΔS° _ads_ were found from the slopes of the curves of ln C_e_ vs 1/T and ln C_e_ vs ln T, respectively. The obtained values are given in Table 5.

**TABLE 5:** Isosteric adsorption enthalpy, ΔH° _ads_ and isosteric adsorption entropy ΔS° _ads_ values.

Temperature (K)	ΔH° _ads_ (kJ/mol)	ΔS° _ads_ (J/molK)
298	−4.93	16.11
313		
343		

From Table 5 it is seen that for a constant adsorbed amount (4.85 mg/g), which roughly corresponds to the formation of a first plateau, the isosteric enthalpy and entropy changes are −4.93 kJ mol^-1^ and 16.11 J mol^−1^ K^−1^, respectively. The rather small and negative enthalpy change indicates that the adsorption is partially exothermic in nature and the interactions are predominantly physical. On the other hand, a very small and positive isosteric entropy change indicates that the dehydration of the dye ions in the hydrated state due to adsorption leads to disorder, that is, partially to an increase in entropy.

### 3.4 pH effect

**FIGURE 5:**
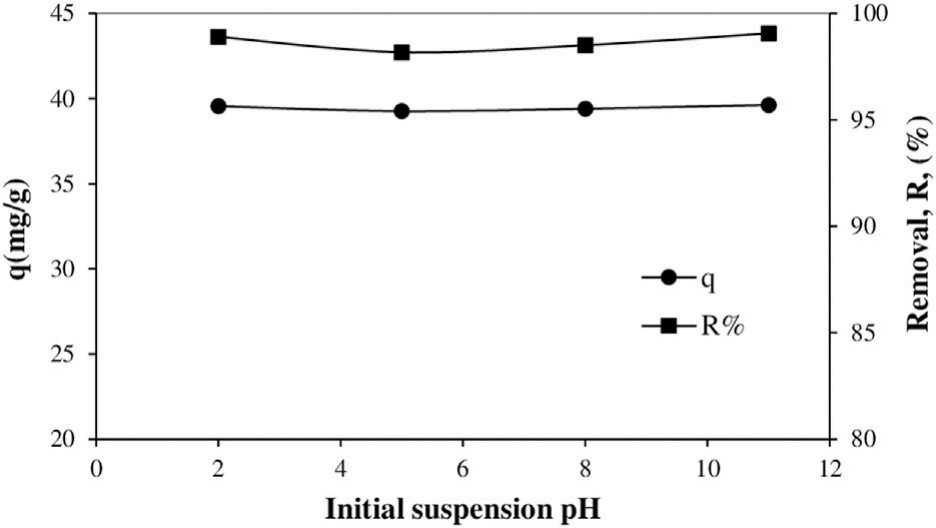
Variation of removal ratios versus the pH of suspension and variation of adsorbed amounts versus the pH of suspension (Initial dye concentration:80 mg/L, temperature:25°C, time: 60 min, solid/liquid ratio:0.2/100 g/m and shaking speed:150 min^−1^).

Solid surfaces, which can have highly charged groups in aqueous suspensions, are particularly sensitive to environmental conditions such as pH, ionic strength and temperature. This usually causes marked changes in the adsorption of ionic substances onto charged solid adsorbents. In this study, the variation of removal ratio and adsorbed amounts with suspension pH are plotted in Figure 5, respectively. As the pH of the aqueous phase increases, a solid surface generally does not become more positive or less negative due to proton adsorption from the solution to the charged sites.

From these graphs, it is seen that there is a partial decrease in the adsorption of anionic dye as pH increases and then partially increases with increasing pH. Since H^+^ and OH^−^ are potential determining ions (Rosen, 1989; Alkan et al., 2005; Gürses et al., 2009), the effect of pH is particularly evident in mineral oxides such as silica and alumina, and an increase in the amount adsorbed means an increase in the removal rate. In addition, the fact that the adsorbed amounts do not change much with increasing pH supports the validity of the proposed multilayer adsorption mechanism, in which the dye ions on the powder pumice surface are adsorbed via pi-pi interactions.

### 3.5 Microscopic and spectroscopic analyzes

In order to support the kinetic, isotherm and thermodynamic based evaluations of the adsorption mechanism of Remazol Red RB on the powder pumice surface and to take into account the structural changes, the FTIR and XRD spectra and HRTEM and static contact angle images taking from the samples after adsorption are given in Figures 7, 8, 9, respectively. The static contact angle values of the raw and post-adsorption samples of powder pumice measured at different initial dye concentrations for water are also shown in Table 6.

**TABLE 6:** Static contact angle values of raw pumice and samples obtained from adsorption experiments performed at different initial concentrations of Remazol Red RB at 25°C.

C_init_ (mg/L)	Contact angle (degree)
0	15.06
10	19.01
20	22.48
30	23.79
40	29.48
50	27.15
60	33.94
70	37.02
80	45.49

**FIGURE 6:**
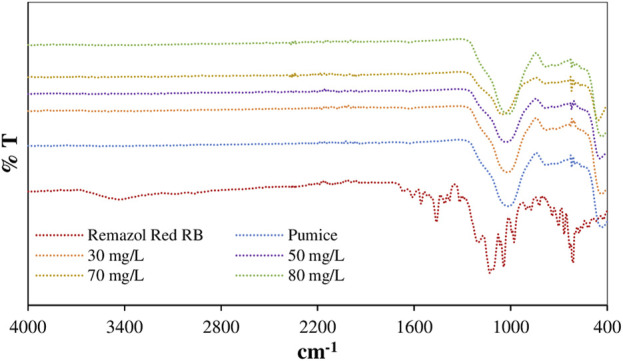
FTIR spectra of raw pumice and samples obtained from adsorption experiments at different dye concentrations at 25°C.

Figure 6 shows the FTIR spectra of raw pumice, Remazol Red RB and samples obtained from adsorption experiments at various initial dye concentrations for wave number range of 4,000–400 cm^−1^.

From this figure, the broad band observed between 1,300 and 820 cm^−1^ can be attributed to the different mineral oxides found in the pumice material (Mourhly et al., 2015). The peaks at 516 and 856 cm^−1^ in the FTIR spectrum of pumice may have resulted from the Si–O bending strength vibrations of the SiO_2_ forming the structure (Ersoy et al., 2010). In addition, the stretch vibration peak of Si–O–Al at 435 cm^−1^, the stretch vibration peak of SI–O–H at 769 cm^−1^, the stretch peak of symmetric and asymmetric Si–O–Si at 1,022 cm^−1^ and the stretch peak of OH at 3,442 cm^−1^ are characteristic for pumice (Moradi et al., 2016; Ramesan et al., 2016). There are specific peaks belonging to functional groups for Remazol Red RB. These are 1,215 and 1,417 cm^−1^ (S=O stretch) for sulfonic acid and sulfite, 1,608 cm^−1^ for N=N stretch vibration, 2,954 cm^−1^ for C-H stretch vibration in CH_2_ groups and 3,446 cm^−1^ for aromatic C-OH stretch vibration (Waghmode et al., 2012). In the spectra of the samples obtained from experiments at high initial dye concentrations, the appearance of characteristic sulfonate and sulfite peaks of the dye implies that the adsorption takes place via pi-pi interactions, not with charged groups.

**FIGURE 7:**
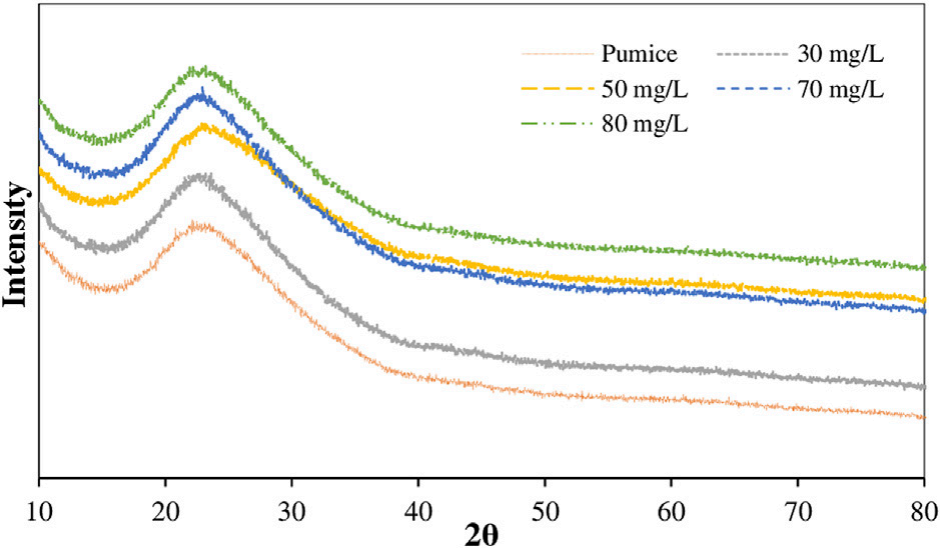
XRD spectra of raw pumice and samples obtained from adsorption experiments at different dye concentrations at 25°C.

Figure 7 shows XRD diffractograms of raw pumice and samples from adsorption experiments at various initial dye concentrations.

From this figure, a broad peak can be seen, which is attributed to the amorphous SiO_2_ of raw pumice and appears between 20° and 30° (2θ) (Azad et al., 2020; Nurwahid et al., 2022). The appearance of a similar broad peak in all other spectra indicates that the dye adsorption did not cause any significant deformation of the crystallographic structure of the pumice and that the adsorption took place at the surface.

**FIGURE 8:**
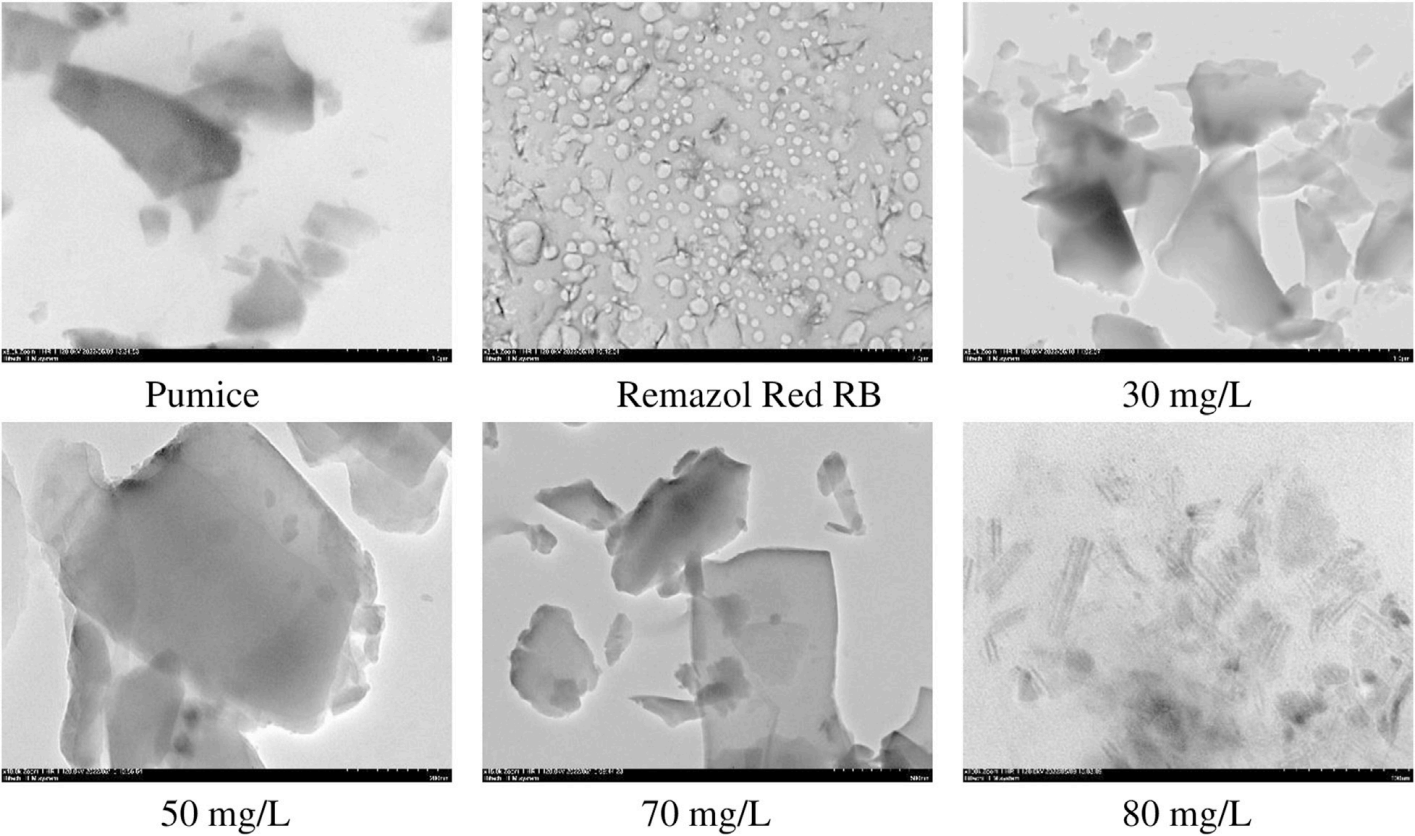
HRTEM images of raw pumice and samples obtained from adsorption experiments at different dye concentrations at 25°C.

The HRTEM images of the samples obtained from the adsorption experiments performed at various initial dye concentrations at 25°C and raw pumice are given in Figure 8.

Raw pumice has a layered structure characterized by the presence of large particles with heterogeneous sizes ranging from 1 µm to a few nanometers (Mourhly et al., 2015). It can be seen from Figure 8 that as the amount of dye adsorbed increases with increasing dye concentration, the layers are separated, causing the plates to appear clearer. In particular, the apparent layer separation and transparency in the image of the sample obtained from the experiment performed at an initial dye concentration of 80 mg/L implies multilayer adsorption of the highly coarse molecular structure dye via pi-pi interactions.

### 3.6 Goniometric analyzes

**FIGURE 9:**
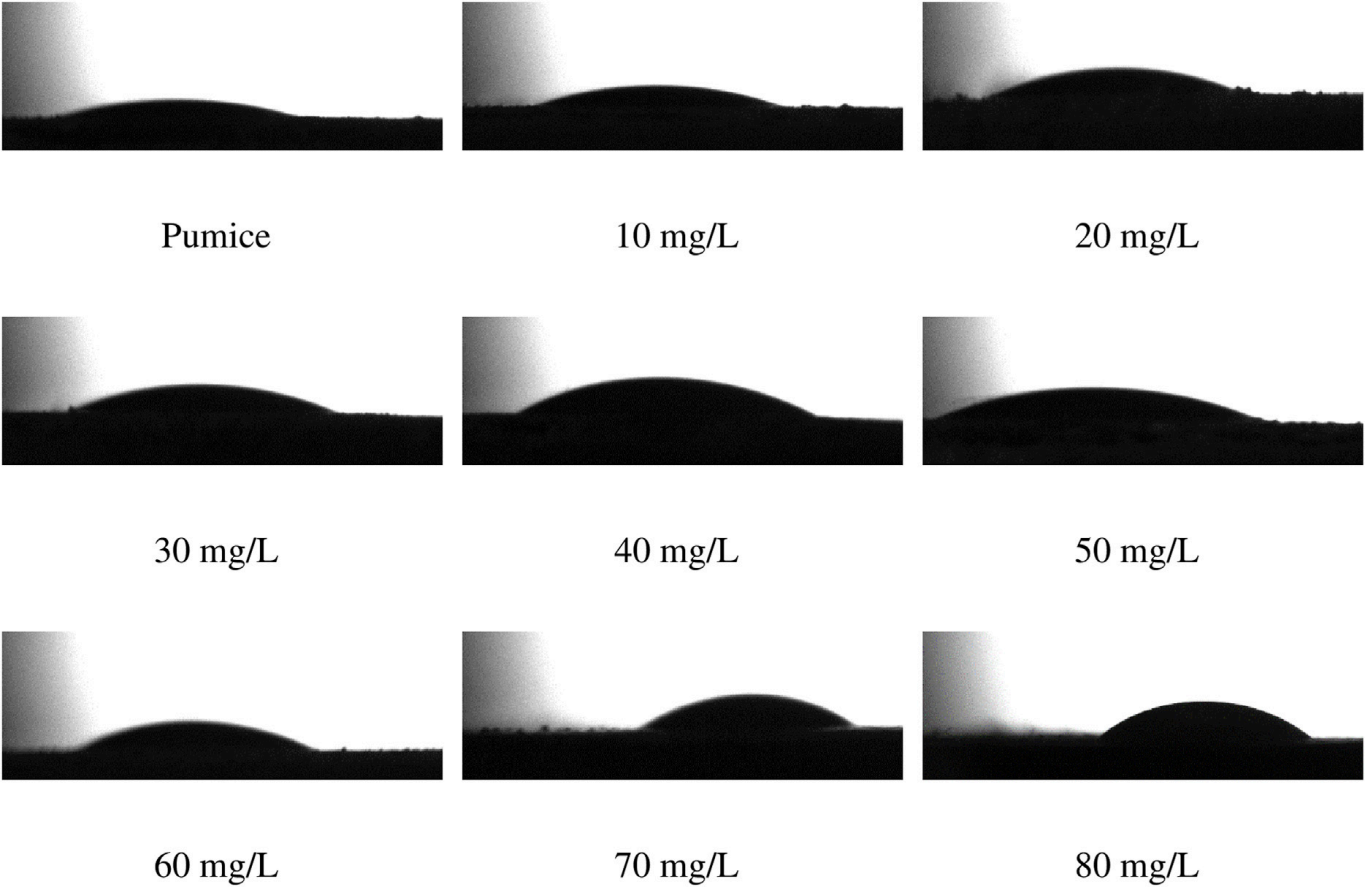
Goniometric images of raw pumice and samples obtained from adsorption experiments at different dye concentrations at 25°C.

Goniometric images and measured static contact angle values of raw pumice and samples obtained from adsorption experiments at different initial Remazol Red RB concentrations at 25°C are given in Figure 9 and Table 6, respectively.

From this figure and table, it is seen that the static contact angle values increase regularly with increasing initial dye concentration and all samples exhibit hydrophilic character. These results may imply that the adsorbate-to-adsorbent interactions are predominantly physical in nature and are not of ion-ion attraction type leading to electro neutralization and are specifically T-shaped pi-pi interactions. It can be argued that multilayer adsorption over pi-pi interactions, especially in samples obtained from experiments at high initial dye concentration, occurs in a conformation of the hydrophobic parts of the dye ion towards the aqueous phase, resulting in an increase in partial hydrophobicity.

### 3.7 Comparison of adsorption capacities

Finally, the maximum adsorption capacity obtained for Remazol Red RB adsorption on the pumice surface in this study was compared with the adsorption capacities reported for similar adsorbent-adsorbate systems in the literature (Table 7).

**TABLE 7:** Comparison of the adsorption capacity obtained for the Pumice-Remazol Red RB system with the adsorption capacities reported for some other similar adsorption systems in the literature.

Adsorbent	Adsorbate	q_max_ (mg/g)	Reference
Nanocrystalline FeNi alloy powder	Remazol Red	80.97	El Boraei et al. (2022)
Ni–Co oxide nano-powder	Remazol Red RB-133	45.25	El Boraei and Ibrahim (2019)
MgO nanomaterials	Remazol Red RB-133	27.3–77.2	Mahmoud et al. (2016)
Fly ash	Remazol Red 133	47.26	Dizge et al. (2008)
Polyaniline/Cerium oxide	Remazol Red RB-133	13.9–18.6	Khairy et al. (2016)
Sludge-based activated carbon	Reactive Red 24	30.49	Li et al. (2011)
Pumice powder	Methylene blue	∼3.1	Akbal (2005)
	Crystal violet	∼2.1	
Pumice stone	Malachite Green	22.57	Shayesteh et al. (2016)
	Crystal Violet	6.99	
Modified pumice stone	Methylene blue	15.87	Derakhshan et al. (2013)
Pumice powder	Remazol Red RB	38.90	This study
Polypyrrole-polyethyleneimine (PPy-PEI) nano-adsorbent	Methylene blue	183.3	Birniwa et al. (2022)
Bentonite clay	Methylene blue	256	Jawad et al. (2023)
Sugar beet-based activated carbon	Methylene blue	185.2	Zayed et al. (2023)
	Methyl orange	140.8	
Pumice modified with H_2_SO_4_	Remazol Black B	10.0	Soleimani et al. (2023)
Raw pumice		5.26	

## 4 Conclusion

This study focused on investigating the kinetics, thermodynamics and adsorption mechanism of Remazol Red RB removal from synthetic wastewater using powdered pumice and the effects of various experimental parameters such as initial dye concentration, time, temperature and pH.

Kinetic studies showed that the pseudo-second-order model can best describe the dye removal kinetics and that intraparticle diffusion control is also effective in adsorption.

Analysis of the fit of the experimental data with various isotherm equations and models for three different temperatures shows that the adsorption of Remazol Red RB on powder pumice is compatible with the Freundlich isotherm equation with the highest correlation coefficient (*R* ^2^). This may indicate the presence of a heterogeneous surface, partially low-efficiency adsorbate-adsorbent interactions and highly effective multilayer adsorption.

The zeta potential values, which were negative at low equilibrium dye concentrations, became positive with increasing equilibrium dye concentration. The measurement results of electrical conductivity values also showed that with increasing equilibrium dye concentration, the electrical conductivity values first increased and then remained relatively constant. Thus, sharp increase in the amount adsorbed with further increase in equilibrium dye concentration can be attributed to the efficiency of non-covalent π–π interactions called π–π stacking, which is either T-shaped or parallel displaced type in adsorption.

Based on the adsorbed amount corresponding to the first plateau formed in the experimental isotherms, the isosteric enthalpy and entropy changes were calculated as −4.93 kJ mol^−1^ and 16.11 J mol^-1^ K^−1^, respectively. The rather small and negative enthalpy change indicates that the adsorption is exothermic in nature and the interactions are predominantly physical. On the other hand, the very small and positive isosteric entropy change can also be attributed to the partial increase in disorder associated with the dehydration of hydrated dye ions by adsorption.

The increase in pH led to a partial decrease in the amount of adsorbed dye, followed by a partial increase. The fact that the adsorbed amounts do not change much with the increase in pH supports the idea that dye ions are adsorbed on the pumice surface via π–π interactions and the validity of the proposed multilayer adsorption mechanism. The characteristic sulfonate and sulfide peaks of the dye in the FTIR spectra of the samples obtained from the experiments performed at high initial dye concentrations were attributed to the fact that the adsorption took place by pi-pi interactions.

The wide peak observed in all XRD spectra may indicate that dye adsorption did not cause a significant deformation in the crystallographic structure of pumice and that adsorption took place on the surface.

It can be seen from the HRTEM images that the amount of adsorbed dye increases with increasing dye concentration, and thus the plates become clearer as the layers are separated.

From the fact that the static contact angles regularly increase with increasing initial dye concentration but all samples are hydrophilic, it can be deduced that the adsorbate-adsorbent interactions are predominantly physical but not of the ion-ion attraction type that leads to electro-neutralization.

## Data Availability

The original contributions presented in the study are included in the article/Supplementary Material, further inquiries can be directed to the corresponding author.
